# Assessing lumbar posture variability in individuals with chronic low back pain in daily life

**DOI:** 10.3389/fbioe.2025.1509634

**Published:** 2025-08-08

**Authors:** Friederike Schömig, Maxim Bashkuev, Sandra Reitmaier, Lena Fleig, Matthias Pumberger, Hendrik Schmidt

**Affiliations:** ^1^ Center for Musculoskeletal Surgery, Charité – Universitätsmedizin Berlin, Berlin, Germany; ^2^ Julius Wolff Institute, Berlin Institute of Health, Charité – Universitätsmedizin Berlin, Berlin, Germany; ^3^ Department of Psychology, Medical School Berlin, Berlin, Germany

**Keywords:** spine, low back pain, movement, conservative treatment, physiotherapy

## Abstract

**Introduction:**

Investigating lumbar spinal posture has become increasingly important for understanding the development and persistence of low back pain (LBP). However, there is a notable lack of studies analyzing changes in lumbar posture variability in individuals with LBP compared with healthy individuals in daily life. This study aims to address this gap by examining whether lumbar posture patterns differ between individuals with and without LBP throughout the day.

**Methods:**

A prospective study design was utilized, including individuals with and without chronic LBP. Lumbar postures were continuously measured over a 24-hour period using the Epionics SPINE system.

**Results:**

The study included 208 (115 females) asymptomatic individuals and 104 (62 females) individuals with LBP. Individuals with LBP exhibited significantly fewer main lumbar postures during the day compared to healthy participants (1.7 vs 1.9 main postures, p = 0.020; r = 0.132). When analyzed by sex, these differences remained statistically significant in males (1.6 vs 1.9 main postures, p = 0.034; r = 0.183) but not in females (1.7 vs 1.8 main postures, p = 0.238; r = 0.089). Age did not significantly influence these results.

**Conclusion:**

The observed changes in lumbar spinal posture variability should be taken into account when developing treatment plans for individuals with chronic LBP. Further research is needed to confirm the impact of increased posture variability on pain persistence.

## 1 Introduction

Low back pain (LBP) stands as a leading cause of years lived with disability, imposing a significant burden on affected individuals and the broader socioeconomic system (GBD, 2015 Disease and Injury Incidence and Prevalence Collaborators et al., 2016). LBP impacts approximately 80% of adults, with 15%–23% of the cases progressing to chronic pain (Freburger et al., 2009; Gore et al., 2012). Despite its prevalence and relevance to healthcare, the underlying mechanisms driving the development and persistence of LBP remain poorly understood, complicating its therapeutic management.

The presence of pain often alters movement behavior, drawing attention to the role of lumbar motion and adaption in the context of chronic pain development (Hodges and Smeets, 2015). Psychological mechanisms, such as pain-related fear and catastrophizing, can lead to the restriction of daily activities and specific movements to minimize pain exposure (Christe et al., 2021). Previous studies have also indicated that LBP is associated with restricted spinal movement and reduced movement variability (Ippersiel et al., 2022; Laird et al., 2014; Lima et al., 2018; Seay et al., 2011; Nzamba et al., 2024).

Movement variability is crucial for normal function, with evidence suggesting that decreased variability and adaptability can increase the loading on specific trunk tissues, potentially leading to tissue damage (Saito et al., 2021; van Dieën et al., 2019). As one important aspect, a sedentary lifestyle has been shown to be associated with an increased risk of developing LBP (Baradaran et al., 2021). However, the concept of variability encompasses various aspects of motion behavior, including movement, coordinative, and kinematic variability, and lacks a clear definition of normal or impaired variability (Saito et al., 2021). In this context, lumbar postures refer to the range of postures and alignments of the lower spine assumed by an individual during daily activities. While several studies have documented changes in different aspects of motion variability in individuals with chronic LBP, the specific differences in motion patterns related to the number of lumbar postures taken during daily activities have not been explored.

This study aimed to quantify spinal motion patterns in individuals with and without LBP, focusing on the variability in lumbar postures taken throughout the day as a promising alternative to laboratory systems in allowing ambulatory motion analysis. By enhancing our understanding of motion changes in individuals with LBP, this research may contribute to the development of more individualized and effective conservative treatment plans. This could significantly impact patient outcomes and reduce the overall burden of LBP on healthcare systems.

## 2 Methods

### 2.1 Participants

Using poster announcements, we prospectively recruited asymptomatic individuals who had not experienced LBP in the previous 6 months, as well as individuals with chronic LBP, defined as pain persisting for at least 12 weeks. For both groups, a body mass index (BMI) of ≤26 kg/m^2^, the presence of a neurological deficit, and a history of spine surgery were exclusion criteria. Data for the asymptomatic cohort was collected between September 2010 and November 2011 and has previously been published by Rohlmann et al. (2014). Data for participants with LBP were collected between September 2022 and March 2023. The study protocol was approved by the institutional Ethics Committee (EA4/011/10, EA1/162/13), and all participants provided written informed consent.

### 2.2 Measurement system and protocol

Lumbar motion was assessed using the Epionics SPINE wearable technology system, which consists of two flexible sensor strips, two tri-axial accelerometers, and a small storage box (Rohlmann et al., 2014; Dr et al., 2016; Taylor et al., 2010). Each sensor strip comprises twelve 25-mm-long segments that measure segment angles at a frequency of 50 Hz via strain-gauge technology. The sensor strips are inserted into special hollow plasters on either side of the spine, 7.5 cm from the midline, with the lowest sensor placed at the level of the posterior superior iliac spine. The accelerometers, located at the base of the sensor strips, measure orientation relative to the earth’s gravitational field. Further information on the system’s structure and validation is provided elsewhere (Schmidt et al., 2024).

As previously described, to standardize the measurement system, participants performed a series of choreographed exercises six times, including upper body flexion, extension, left and right axial rotation while standing with extended knees (Rohlmann et al., 2014). Each participant watched a video demonstrating the exercise sequence before performing the tasks. After completing the exercises, participants continued with their daily activities for 24 h, avoiding showering or bathing during this period.

### 2.3 Data analysis

Data analysis followed previously established protocols (Rohlmann et al., 2014). The thoracolumbar lordosis angle (LA) in the relaxed standing position was determined by calculating the median LA of all standing positions measured during the standardized motion sequence. The LA was defined as the sum of the six most caudal segments of the sensor strips, averaged between left and right sides. Symmetrical motion in the sagittal plane with similar values for the left and right sensors indicated pure flexion or extension of the upper body. Asymmetric motion resulted in different LAs on the left and right sides. We analyzed exactly 24 h of data, filtering the raw data from the sensor strips using an eighth-order low-pass Butterworth filter with a cut-off frequency of 5 Hz.

### 2.4 Statistical analysis

Data normality was assessed using the Kolmogorov-Smirnov test. Nominal data were compared using the Chi-Squared test, while unpaired parametric data were analyzed with Student’s t-test. Ordinal data comparisons were conducted using the Mann-Whitney-U test. The correlation between age and the number of lumbar postures were examined using Kendall-Tau-c. Statistical significance was defined as a p-value of <0.05. Effect sizes were categorized as follows: 0.1–0.3 as small, 0.3–0.5 as medium, and >0.5 as large. Statistical analyses were performed using SPSS Version 27.

## 3 Results

A total of 393 participants were measured, 81 of which were excluded due to a BMI >26 kg/m^2^. Thus, 312 participants (208 asymptomatic participants; 104 individuals with LBP) were included into the analysis. Demographic data for the two groups is shown in Table 1.

**TABLE 1:** Demographic parameters of the included participants with and without low back pain (LBP). For age and body mass index (BMI), mean values with standard deviation are given.

	Asymptomatic participants (n = 208)	Individuals with LBP (n = 104)	p-value
Age (years)	40.3 ± 14.0	50.9 ± 13.2	<0.001^a^
Sex (f:m)	115:93	62:42	0.545
BMI (kg/m^2^)	22.6 ± 2.0	23.1 ± 2.3	0.091

^a^ indicates statistically significant differences.

**FIGURE 1:**
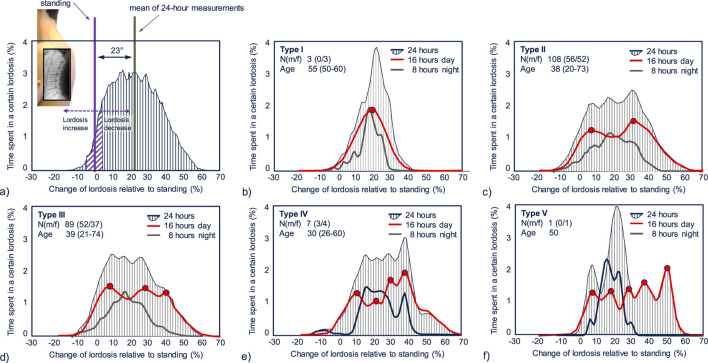
**(a)** Time spent in a certain lordotic posture over 24 h–average curve of 208 asymptomatic subjects. A lumbar lordosis of 0° corresponds to the value in upright standing measured during standardized short-term measurements. Values > 0° indicate reduced lordosis, and values < 0° indicate increased lordosis. Approximately 9% of the day was spent in a lordosis ranging over ±5° of the lordosis measured during standing (violet diagonal-striped). Approximately 90% of the day was spent in a lordosis that was more than 5° smaller or larger than the standing lordosis (vertically striped). **(b–f)** Division into five types based on the number of peaks in the lordosis distribution during 16 h of daily activities.

Analyses of our asymptomatic cohort revealed five distinct lordosis distributions over a 16-hour daytime period, characterized by varying numbers of peaks (Figure 1). Each peak corresponds to a specific global body posture. Among the study participants, 50% exhibited a bimodal pattern. Activities performed while standing (first peak) were clearly distinguishable from those performed while sitting (second peak). One percent exhibited a unimodal pattern due to predominantly sitting activities. Participants with tri- or multimodal distributions, featuring four or more peaks, engaged in a broader range of daytime activities, leading to substantially different postures.

The LBP cohort exhibited four distinct lordosis distributions over a 16-hour daytime period. Among them, 43% showed a bimodal pattern, followed by 45% with a trimodal pattern. Six percent exhibited a unimodal pattern, and 6% displayed higher numbers of peaks.

**FIGURE 2:**
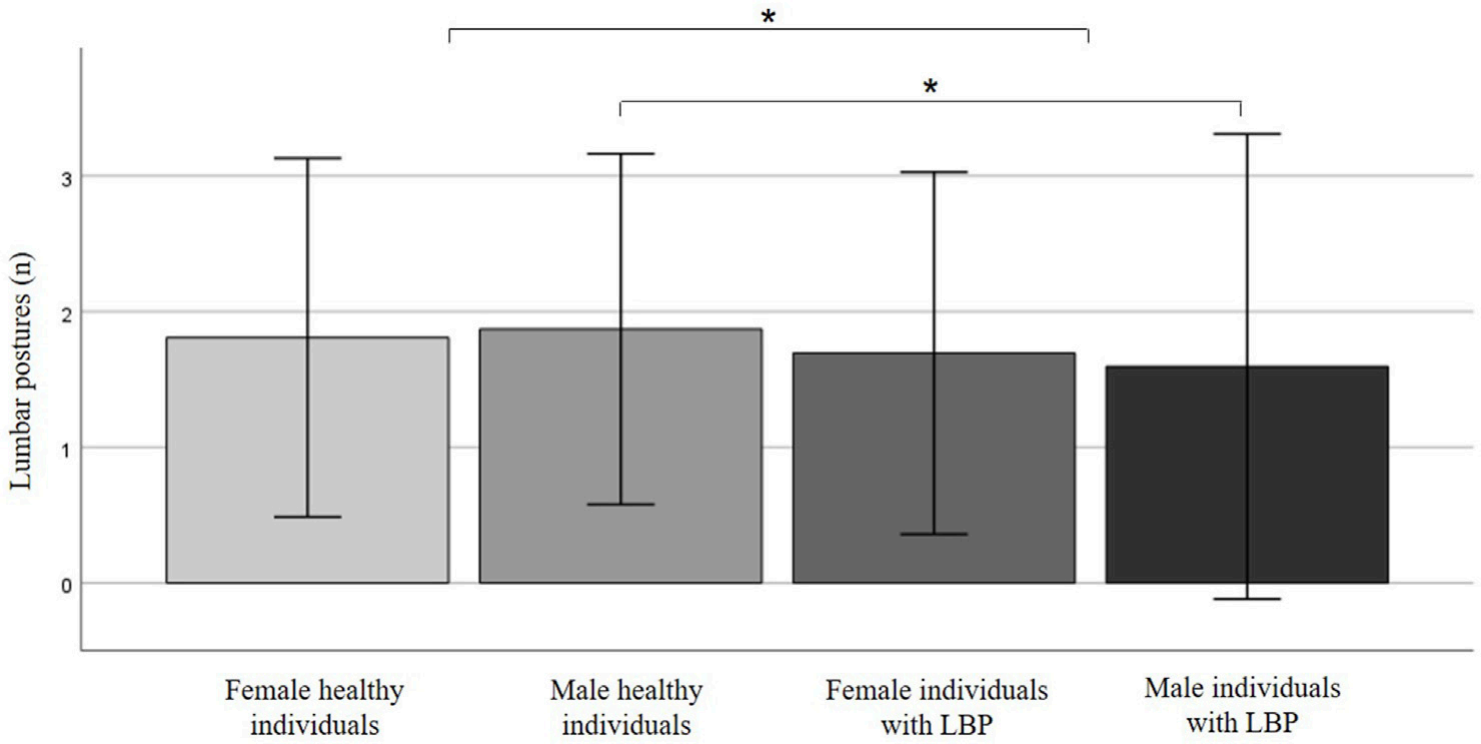
Number of primary lumbar postures assumed over a 16-hour daytime period in asymptomatic individuals and individuals with low back pain (LBP), stratified by sex. Data analysis was performed using the Mann-Whitney-U test. * marks statistically significant differences.

Individuals with LBP showed significantly fewer lumbar postures during the day compared to healthy participants (1.7 vs 1.9 postures, p = 0.020; z = −2.329; r = 0.132) (Figure 2). When grouped for sex, females with LBP did not show significantly fewer postures during the day compared to healthy females (1.7 vs 1.8 postures, p = 0.238; z = −1.180; r = 0.089) while males with LBP did show significantly fewer postures during the day compared to healthy males (1.6 vs 1.9 postures, p = 0.034; z = −2.125; r = 0.183).

There was no significant difference between female and male participants regarding the number of postures taken during the day within the group of healthy participants (female 1.8 vs male 1.9 postures, p = 0.436; z = −0.779; r = 0.054) or within the LBP group (female 1.7 vs male 1.6 postures, p = 0.461; z = −0.736; r = 0.072).

There was no significant correlation between age and the number of lumbar postures assumed during the day in both individuals with LBP (r = −0.064, p = 0.439) or asymptomatic individuals (r = −0.069, p = 0.200).

## 4 Discussion

Despite the clinical relevance of both preventing and treating LBP, little is known about the differences in main lumbar postures taken in daily life (as opposed to laboratory settings) between individuals with or without chronic LBP. This study aimed to analyze the number of lumbar postures taken during the day in people’s natural environments to better understand the differences in posture variability between these groups. Our results indicate that individuals with LBP take significantly fewer lumbar postures throughout the day compared to asymptomatic individuals. This difference was observed in males but not in females. Additionally, within the groups of individuals with LBP and asymptomatic participants, no significant differences were found between male and female participants.

These results are in line with previous studies indicating that movement patterns alter in the presence of pain (Hodges and Smeets, 2015; Nzamba et al., 2024). Various mechanisms have been proposed to explain the reduction in movement associated with pain. While initial adaptations of movement serve as a protective function to prevent further damage to the painful tissue and may benefit the patient initially, psychological factors, such as fear avoidance, play an important role in chronicity of movement and function reductions in painful states (Christe et al., 2021; Vlaeyen and Linton, 2000).

To date, most analyses of spinal motion behavior have focused on spinal range of motion rather than specific spinal motion patterns (Arshad et al., 2019; Pan et al., 2020). Although pain has been shown to affect motion variability, studies examining this variability in the context of back pain have primarily investigated changes in kinematic variability during repetitive functional tasks or pathological coordination (Seay et al., 2011; Saito et al., 2021; Alsubaie et al., 2023). In this context, individuals with LBP exhibit a more “guarded gait” with decreased coordination variability, which has previously been interpreted as reduced adaptability of the locomotor system (Seay et al., 2011; Selles et al., 2001). However, there is currently no established definition of “normal” motion variability as assessed in a person’s daily life.

Spinal postures throughout the day exhibit significant variability, influenced by a range of activities and individual physiological characteristics. Understanding these variations in daily life is crucial, as they can impact spinal health and overall biomechanical function. Research indicates that sedentary behavior, particularly prolonged sitting, is associated with sustained flexed postures, which may contribute to the development of LBP (Baradaran et al., 2021). Conversely, dynamic activities such as walking or standing engage different spinal segments, promoting a variety of postures that could enhance spinal flexibility and reduce stiffness. The interplay between static and dynamic postures is essential in maintaining spinal health, as it facilitates the distribution of mechanical loads across different structures, potentially mitigating the risk of degenerative changes (Hasegawa et al., 2018). Furthermore, the adaptation of spinal postures in response to pain demonstrates a complex interaction between biomechanical and neuromuscular control mechanisms. Individuals with LBP often exhibit altered postural patterns, characterized by reduced movement variability and protective postures, which, while initially beneficial in preventing further tissue damage, may lead to chronic dysfunction if not properly managed (Saito et al., 2021). Therefore, a comprehensive analysis of daily spinal posture variability can provide insights into the prevention and management of LBP, emphasizing the need for balanced activity patterns and the integration of movement into daily routines to support spinal health (Harbourne and Stergiou, 2009).

Our study revealed that overall, individuals with LBP exhibit significantly fewer main lumbar postures during the day compared to asymptomatic participants. This reduction in movement variability among individuals with LBP suggests a possible link between limited postural diversity and the presence of LBP. When examining the data by sex, a notable difference emerged. Female individuals with LBP did not show significantly fewer postures during the day compared to healthy females. In contrast, males with LBP demonstrated significantly fewer postures compared to healthy males. This sex-specific difference suggests that males with LBP might be more prone to adopting fewer lumbar postures, which could be due to varying pain coping mechanisms or differences in daily activities between sexes. However, within each group—healthy participants and individuals with LBP—there were no significant differences between male and female participants in the number of postures taken during the day. This indicates that sex alone does not significantly contribute to the number of lumbar postures in either healthy or LBP populations when considered independently.

Additionally, our analysis found no significant correlation between age and the number of lumbar postures assumed during the day in both individuals with LBP and asymptomatic individuals. This suggests that, in the present sample, age does not significantly affect lumbar movement variability in either group, highlighting that the presence of LBP rather than age might be a more critical factor modulating posture diversity.

Some limitations of our study need to be discussed. Due to the observational study design, statements regarding the results’ causality are not possible. Furthermore, as this study is the first to analyze 24-h measurements of spinal postures, a traditional sample size calculation was not possible. There was no existing literature to predict effect sizes or patterns from. We do, however, anticipate our data to be important for future sample size calculations in similar study designs. Additionally, spinal motion was not measured directly on the spine. However, previous studies have found that measuring posture on the back correlates with measurements performed on the spine and that the Epionics SPINE has a high agreement with a motion capture system (Adams et al., 1986; Stokes et al., 1987; Suter et al., 2020). As data on the participants’ occupation was not collected, an analysis regarding the amount of physical activity during work was not possible. Furthermore, the differences in age between the two groups limited our statistical analysis. Lastly, individuals with a BMI of >26 kg/m^2^ were excluded to avoid bias due to soft tissue. Therefore, we cannot make any conclusions regarding obese individuals.

### 4.1 Conclusion

Overall, our findings underscore the importance of considering sex-specific differences in lumbar movement patterns among individuals with LBP. The observed reduction in postural variability, particularly among males with LBP, may contribute to the persistence of pain and warrants further investigation. These insights emphasize the need for targeted interventions that address movement diversity, potentially enhancing rehabilitation strategies for individuals with LBP. Future research should explore the underlying mechanisms driving these sex differences and the role of specific daily physical activities in shaping lumbar posture variability.

## Data Availability

The raw data supporting the conclusions of this article will be made available by the authors, without undue reservation.
